# A rare case of Merkel cell carcinoma presenting as a giant intra-thoracic mass: A case report and review of the literature

**DOI:** 10.1097/MD.0000000000008743

**Published:** 2017-11-17

**Authors:** Feng-Wei Kong, Miao Zhang, Heng Wang, Cun-Tao Lu, Wen-Bin Wu, Yuan-Yuan Liu

**Affiliations:** a Department of General Surgery, Xuzhou Infectious Disease Hospital; b Department of Thoracic Surgery; c Department of Respiratory Medicine, Xuzhou Central Hospital Affiliated to Southeast University, Xuzhou, China.

**Keywords:** ectopic, extensive radical resection, Merkel cell carcinoma (MCC), neuroendocrine tumor, oligometastasis

## Abstract

**Rationale:**

Merkel cell carcinoma (MCC) is an aggressive neuroendocrine-derived cutaneous cancer. Ectopic or single metastatic MCC located in thorax is extremely rare; meanwhile, its definite management has not been elucidated yet.

**Patient concerns:**

A 64-year-old female patient with a giant mass located in her left thorax was presented for stuffy pain of left chest for 6 months and fever for half a month. She underwent radical resection of vulvar MCC 10 years ago.

**Diagnoses:**

Computed tomography (CT)-guided biopsy of the intrathoracic mass revealed a diagnosis of MCC, without synchronous urogenital lesions on pelvic CT images.

**Interventions:**

This bulky tumor was completely resected via thoracotomy, along with the adjacent pulmonary lobe, pericardium, pleura, and diaphragm.

**Outcomes:**

The patient survived without local-regional recurrence or distant metastasis during the follow-up of 1 year up to now.

**Lessons:**

Ectopic or single metastatic MCC should be considered in the differential diagnosis of intrathoracic tumors, especially in patients with a history of MCC. Besides, a timely surgery combined with chemotherapy is effective for this disease.

## 1 Introduction

Merkel cell carcinoma (MCC) is a rare and aggressive neuroendocrine tumor that occurs predominantly in the head and neck region, which is preferentially occurs in elderly and immunosuppressed patients with a high mortality.^[1]^ Accurate differential diagnosis and staging of MCC depend on biopsy using immunohistochemical staining. Its aggressive nature results in the necessity of multidisciplinary treatment.^[2]^ Surgery combined with adjuvant radiotherapy is effective for resectable cases, while definitive radiotherapy could be considered for unresectable lesions, because MCC is highly sensitive to radiotherapy.^[3]^ However, there are still no approved treatments for patients with metastatic MCC. Herein, an extremely rare case with ectopic (or single metastatic) MCC merely located in thorax was presented for discussion, followed by critical review of related literature, with the aim of promoting the identification and management of MCC.

## 2 Case presentation

A 64-year-old female patient was admitted for moderate stuffy pain of the left chest for 6 months, and productive cough with fever in the recent half a month, without significant loss of weight. Her highest temperature during this period was 38.5°C. She was initially diagnosed as pneumonia in a community hospital, but the therapy using antibiotics of piperacillin-tazobactam for 1 week did not work. She had been working near a coal mine for more than 10 years, without history of hypertension or coronary heart disease. Her family and social histories were unremarkable. The patient underwent extensive radical resection of vulvar cutaneous MCC 10 years ago, and the surgical margin as well as inguinal lymph nodes were tumor-negative. She took cranial magnetic resonance, chest and abdomen computed tomography (CT) annually thereafter, which excluded tumor recurrence or metastasis in the past 9 years. Thorough physical examination failed to identify any suspicious lesions.

**Figure 1:**
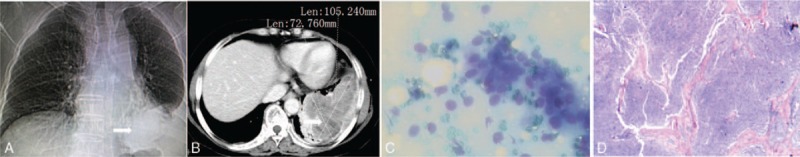
A, Chest X-ray of the patient on admission showed an irregular, giant mass in her left lower thorax. B, CT demonstrated a bulky tumor adjacent to thoracic aorta, with suspicious involvement of mediastinum, pericardium, pleura, and diaphragm. C, CT-guided biopsy showed an aggregation of atypical malignant cells. D, Postoperative histological test confirmed MCC, by H–E staining (×200). CT = computed tomography, MCC = Merkel cell carcinoma.

Related tests were carried out step by step for differential diagnosis. Laboratory tests for hepatic and renal functions were normal. Besides, repeated culture of her sputum for tuberculosis, fungus, parasite, or bacteria turned out to be negative. Diabetes mellitus and rheumatism were also excluded. Additionally, serum tumor markers of carcinoembryonic antigen, cytokeratin 19 fragment, squamous cell carcinoma, neuron specific enolase, alpha fetal protein, serum ferritin, β-human chorionic gonadotrophin (β-HCG), carbohydrate antigen 242 (CA242), CA72-4, CA153, CA125, and CA19-9 were all in normal range. Radiological examinations were performed, because her symptoms could not be explained by the above findings. Chest X-ray and CT revealed an irregular mass approximately 15.0 cm × 15.0 cm × 7.0 cm in size (Fig. 1A), which was located in the left thorax adjacent to the thoracic aorta. Atelectasis of left lower pulmonary lobe, enlarged mediastinal lymph nodes were also indicated (Fig. 1B). The cranial magnetic resonance image (MRI) and bone emission CT excluded detectable distant metastasis or other suspicious lesions. Positron emission tomography was not performed because it was not covered by her health insurance. CT-guided fine needle aspiration biopsy of the mass showed aggregation of atypical malignant cells (Fig. 1C). Further immunohistochemistry staining indicated positive expression of CD99, CD56, vimentin and Ki67 (20%), which indicated typical MCC. Thus, her diagnosis was corrected as locally advanced ectopic or single metastatic MCC with obstructive pneumonia, which could explain the complaints of chest pain and fever.

Radical surgery combined with adjuvant chemotherapy was assumed to be feasible after multidisciplinary consultation with specialists in medical oncology, gynaecology, and cardiothoracic surgery. The Eastern Cooperative Oncology Group Performance Status of the patient was grade 1. Preoperative tests of cardiopulmonary functions were carried out. Routine electrocardiogram, ultrasonic cardiogram indicated nothing abnormal. The forced expiratory volume in 1 second was 2.42 L, while the maximum voluntary ventilation was 73.4%. In addition, her left ventricular ejection fraction was 60%, which showed that this patient could tolerate thoracotomy. Preoperative surgical plan using three-dimensional (3D) printing model of this complicated tumor was not performed because of financial reason. The resection extent was decided to be along with the morphologically involved adjacent parietal pleura, pericardium, and diaphragm according to the principles of oncological surgery.

Antibiotics of cefatriaxone combined with levofloxacin were administered for 7 days before the surgery, and her temperature was reduced to 37°C. Then thoracotomy was performed through the 5th intercostal space, under general anesthesia and double-lumen endotracheal intubation. After the collapse of the left pulmonary lobes, fortunately, the significant distance between the tumor and thoracic aorta emerged. Meanwhile, adhesion of the mass with adjacent pericardium and pleura was observed during the surgery. Then a single-stage operation including left lower lobectomy, extensive resection of the mass, and mediastinal lymph node dissection was performed successfully, which took 290 minutes. Postoperative pathological staining of the mass revealed neuroendocrine carcinoma (Fig. 1D), which involved the left lower pulmonary lobe, adjacent pericardium, diaphragm, and pleura. The surgical margins and dissected lymph nodes were tumor-negative. In addition, the feeding vessels of the tumor were verified to be branches of internal thoracic vessels. Further immunohistochemical staining suggested positive expression of CD56 and Ki67 (25%), and negative chromogranin A, synaptophysin, CD10, P63, CK5/6, human leukocyte common antigen, vimentin and desmin, which revealed MCC.

Based on these findings, a diagnosis of intrathoracic ectopic, or single metastatic MCC (oligometastasis) was established, because her physical examination and pelvic CT did not indicate synchronous vulvar lesions. The patient was staged as IIB tentatively according to the 8th AJCC consensus guidelines for MCC (primary tumor invaded fascia, muscle, cartilage or bone, without regional lymph node metastases).^[4]^ However, it was not a definite diagnosis for insufficient histological evidence.

The postoperative recovery of the patient was mainly uneventful. Her body temperature returned to normal after the administration of piperacillin-tazobactam for another 7 days, and her chest pain was alleviated gradually. She was discharged 14 days after the surgery. Subsequently, she received 4 cycles of adjuvant chemotherapy with a mean interval of 21 days, using carboplatin (area under the curve [AUC] = 5) on day 1, and etoposide 100 mg daily on day 1 to 4. This patient survived without loco-regional recurrence or distant metastasis during the follow-up of 1 year.

## 3 Discussion

The clinical thinking process of this case in terms of safety and etiological analysis should be criticized. First, the symptoms of chest pain and fever might be correlated with many diseases, such as acute bronchitis, empyema, arteritis, pneumonia, rheumatic fever, tuberculosis, pericarditis, myocarditis, coronary artery disease, lung cancer, heart attack, angina, acid reflux (gastro-esophageal reflux disease), esophagitis, cor pulmonale, pulmonary embolism, and pleurisy. As for this case, hemorrhage, edema, or necrosis within the intrathoracic MCC and atelectasis of the adjacent lobe may lead to atypical fever and chest pain, which were alleviated lastly after the surgery and the administration of perioperative antibiotics. However, there is no clear evidence available that atelectasis is associated with fever.^[5]^ Based on the complaints of this patient on admission, the initially suspected diseases need to be distinguished step by step, including acute coronary syndrome, coronary heart disease, aortic dissection, pulmonary embolism, silicosis, atelectasis, and pneumonia with specific infection by tuberculosis, fungus or parasites, rheumatic disease, recurrence of the MCC, and newly emerged malignancy. Related work-up and examinations of neurogenic mediastinal and chest malignancies including the tumor markers, serum cardiac enzymes such as lactate dehydrogenase, creatine phosphokinase, creatine kinase-MB, and alpha-hydroxybutyric dehydrogenase should be taken, which is helpful for differential diagnosis.

Second, anterior, middle, and posterior mediastinal tumors should be taken into account before surgery, which could invade into the thorax and mimic intrathoracic tumor. MRI and biopsy are useful for the diagnosis of mediastinal masses. Because of the proximity of thoracic aorta to MCC and potential adhesion between each other, the preoperative assessment of aorta with surgical plan is necessary. In detail, an aortic specialist surgeon with preparation for thoracic endovascular aortic repair ought to participate in this operation according to the basic standards of clinical care, because the patient would take the risk of accidental aortic rupture during the surgery.

The incidence of MCC is increasing for unknown reasons, which is estimated to be 0.32/100,000 in the United States.^[6]^ MCC has a high rate of sentinel lymph node involvement, local-regional recurrence, and mortality.^[7]^ It is reported that 65% of MCC patients are admitted with local disease, whereas 26% and 8% are presented with nodal and distant disease, respectively.^[3]^ Risk factors for the onset of MCC include Merkel cell polyomavirus (MCPyV) infection, ultraviolet light exposure, history of chronic inflammatory disorders, immunosuppression, and advanced age.^[2,8]^ MCPyV infection is highly prevalent in the skin of the general population, which has been identified in up to 80% of MCC cases, especially in elderly and immunosuppressed patients.^[9,10]^ Approximately 20% of MCCs are not driven by MCPyV (virus-negative), which can be identified by immunohistochemistry using the CM2B4 antibody, and they represent a more aggressive subtype.^[11]^ The tumorigenesis of virus-negative MCC is linked to UV-induced DNA damage.^[12]^ Neuroendocrine MCC with highly aggressive behavior rarely arise in the vulva,^[13]^ which originates from mechanoreceptor Merkel cells of the stratum basale of epidermis.^[14]^ However, the evidence regarding the actual origin and etiology of MCC is insufficient due to its rarity.^[13]^ The presented patient with ectopic or second MCC in our report has performed radical resection of vulvar MCC 10 years ago, which indicates that long-term follow-up is mandatory. Although MCC is chemosensitive and radiosensitive, responses in advanced stages are mostly of short duration.

There are still no approved treatments for patients with metastatic MCC.^[2]^ The treatment choice depends on stage at presentation, regional lymph node status, and comorbidities of the patient. Surgical resection with clear margins and lymph nodes dissection, followed by adjuvant radiotherapy, is recommended for MCC patients with local-regional nodal involvement.^[2,15]^ Reconstructed models of sophisticated anatomical structures by means of 3D rapid prototyping, which provide better visualization, can be used to facilitate surgical planning and to assess the invasion of vital structures such as thoracic aorta by adjacent tumors.^[16]^

As for the patient in this study, preoperative simulation using 3D printing of this complicated tumor might be more helpful than CT images to understand its location, and relationship with adjacent thoracic aorta. Meanwhile, a timely extensive radical resection of MCC could also deliver satisfactory disease control.

Definitive radiotherapy for localized MCC may confer regional control.^[17]^ Radiotherapy alone, or in combination with chemotherapy, can be considered for unresectable MCC; meanwhile, chemotherapy remains the most commonly used regimen for distant metastatic MCC.^[2]^ The tumor should be excised with 1–2 cm margins, and radical lymphadenectomy should be performed simultaneously if the regional lymph node is involved.^[15]^ Wide local excision, selective lymphadenectomy, and loco-regional radiotherapy are now served as the mainstay of treatment for aggressive primary MCC, with acceptable loco-regional disease control. Chemotherapy is reserved for advanced-stage MCC and palliative therapy.^[18]^ The discovery of molecular alterations and MCPyV of MCC lead to novel targeted therapy and immunotherapy trials.^[12,19]^ Checkpoint inhibitors are emerging therapeutic options.^[20]^ Immunotherapy targeting the programmed cell death receptor 1/programmed cell death ligand 1 (PD-1/PD-L1) checkpoint may provide durable responses for advanced MCC.^[18]^ Avelumab is a promising new therapeutic agent for patients with metastatic Merkel cell carcinoma.^[21]^ Furthermore, the metastatic lesions of MCC demonstrated avidity to both somatostatin receptors and (18)F-FDG, and an excellent partial response to a single cycle of peptide receptor radionuclide therapy with (177)Lu-DOTATATE is demonstrated, which may be reasonable as the first-line therapy for metastatic MCC.^[22]^ The specific biomarkers of MCC include platelet-derived growth factor receptor, CD117, phosphoinositide 3-kinase, PD-L1, p63, vascular endothelial growth factor receptor, Ki-67, CD34, epithelial cellular adhesion molecule (Ep-CAM), nuclear factor kappa B, sonic hedgehog pathway proteins, and matrix metalloproteinase, which have been suggested to predict the prognosis of these patients.^[2]^ Up to date, no effective systemic treatment for end stage MCC is available.^[23]^ Therefore, more high-quality studies are needed for the establishment of the optimal therapeutic regimens, and detailed indications of aggressive surgery for these patients.

## 4 Conclusion

In summary, ectopic or single metastatic MCC should be kept in mind during the differential diagnosis of newly emerged intrathoracic masses, especially for patients with an MCC history. Moreover, single-stage extensive radical resection of MCC combined with chemotherapy is effective for selected patients.
